# Incident HIV during Pregnancy and Postpartum and Risk of Mother-to-Child HIV Transmission: A Systematic Review and Meta-Analysis

**DOI:** 10.1371/journal.pmed.1001608

**Published:** 2014-02-25

**Authors:** Alison L. Drake, Anjuli Wagner, Barbra Richardson, Grace John-Stewart

**Affiliations:** 1 Department of Global Health, University of Washington, Seattle, Washington, United States of America; 2 Department of Epidemiology, University of Washington, Seattle, Washington, United States of America; 3 Department of Biostatistics, University of Washington, Seattle, Washington, United States of America; 4 Vaccine and Infectious Diseases Division, Fred Hutchinson Cancer Research Center, Seattle, Washington, United States of America; 5 Department of Medicine, University of Washington, Seattle, Washington, United States of America; 6 Department of Pediatrics, University of Washington, Seattle, Washington, United States of America; National Institute of Child Health and Human Development, United States of America

## Abstract

**Background:**

Women may have persistent risk of HIV acquisition during pregnancy and postpartum. Estimating risk of HIV during these periods is important to inform optimal prevention approaches. We performed a systematic review and meta-analysis to estimate maternal HIV incidence during pregnancy/postpartum and to compare mother-to-child HIV transmission (MTCT) risk among women with incident versus chronic infection.

**Methods and Findings:**

We searched PubMed, Embase, and AIDS-related conference abstracts between January 1, 1980, and October 31, 2013, for articles and abstracts describing HIV acquisition during pregnancy/postpartum. The inclusion criterion was studies with data on recent HIV during pregnancy/postpartum. Random effects models were constructed to pool HIV incidence rates, cumulative HIV incidence, hazard ratios (HRs), or odds ratios (ORs) summarizing the association between pregnancy/postpartum status and HIV incidence, and MTCT risk and rates. Overall, 1,176 studies met the search criteria, of which 78 met the inclusion criterion, and 47 contributed data. Using data from 19 cohorts representing 22,803 total person-years, the pooled HIV incidence rate during pregnancy/postpartum was 3.8/100 person-years (95% CI 3.0–4.6): 4.7/100 person-years during pregnancy and 2.9/100 person-years postpartum (*p* = 0.18). Pooled cumulative HIV incidence was significantly higher in African than non-African countries (3.6% versus 0.3%, respectively; *p*<0.001). Risk of HIV was not significantly higher among pregnant (HR 1.3, 95% CI 0.5–2.1) or postpartum women (HR 1.1, 95% CI 0.6–1.6) than among non-pregnant/non-postpartum women in five studies with available data. In African cohorts, MTCT risk was significantly higher among women with incident versus chronic HIV infection in the postpartum period (OR 2.9, 95% CI 2.2–3.9) or in pregnancy/postpartum periods combined (OR 2.3, 95% CI 1.2–4.4). However, the small number of studies limited power to detect associations and sources of heterogeneity.

**Conclusions:**

Pregnancy and the postpartum period are times of persistent HIV risk, at rates similar to “high risk” cohorts. MTCT risk was elevated among women with incident infections. Detection and prevention of incident HIV in pregnancy/postpartum should be prioritized, and is critical to decrease MTCT.

*Please see later in the article for the Editors' Summary*

## Abstract 2

Alison Drake and colleagues conduct a systematic review and meta-analysis to estimate maternal HIV incidence during pregnancy and the postpartum period and to compare mother-to-child HIV transmission risk among women with incident versus chronic infection.

*Please see later in the article for the Editors' Summary*

## Editors' Summary

**Background:**

Worldwide, about 3.4 million children younger than 15 years old (mostly living in sub-Saharan Africa) are infected with HIV, the virus that causes AIDS by gradually destroying immune system cells, thereby leaving infected individuals susceptible to other serious infections. In 2012 alone, 230,000 children (more than 700 every day) were newly infected with HIV. Most HIV infections among children are the result of mother-to-child HIV transmission (MTCT) during pregnancy, delivery, or breastfeeding. The rate of MTCT (and deaths among HIV-positive pregnant women from complications related to HIV infection) can be greatly reduced by testing women for HIV infection during pregnancy (antenatal HIV testing), treating HIV-positive women with antiretroviral drugs (ARVs, powerful drugs that control HIV replication and allow the immune system to recover) during pregnancy, delivery, and breastfeeding, and giving ARVs to their newborn babies.

**Why Was This Study Done?:**

The World Health Organization and the Joint United Nations Programme on HIV/AIDS (UNAIDS) have developed a global plan that aims to move towards eliminating new HIV infections among children by 2015 and towards keeping their mothers alive. To ensure the plan's success, the incidence of HIV (the number of new infections) among women and the rate of MTCT must be reduced by increasing ARV uptake by mothers and their infants for the prevention of MTCT. However, the risk of HIV infection among pregnant women and among women who have recently given birth (postpartum women) is poorly understood because, although guidelines recommend repeat HIV testing during late pregnancy or at delivery in settings where HIV infection is common, pregnant women are often tested only once for HIV infection. The lack of retesting represents a missed opportunity to identify pregnant and postpartum women who have recently acquired HIV and to prevent MTCT by initiating ARV therapy. In this systematic review (a study that uses predefined criteria to identify all the research on a given topic) and meta-analysis (a study that uses statistical methods to combine the results of several studies), the researchers estimate maternal HIV incidence during pregnancy and the postpartum period, and compare the risk of MTCT among women with incident (new) and chronic (long-standing) HIV infection.

**What Did the Researchers Do and Find?:**

The researchers identified 47 studies (35 undertaken in Africa) that examined recent HIV acquisition by women during pregnancy and the 12-month postpartum period. They used random effects statistical models to estimate the pooled HIV incidence rate and cumulative HIV incidence (the number of new infections per number of people at risk), and the association between pregnancy/postpartum status and HIV incidence and MTCT risk and rates. The pooled HIV incidence rate among pregnant/postpartum women estimated from 19 studies (all from sub-Saharan Africa) that reported HIV incidence rates was 3.8/100 person-years. The pooled cumulative HIV incidence was significantly higher in African countries than in non-African countries (3.6% and 0.3%, respectively; a “significant” difference is one that is unlikely to arise by chance). In the five studies that provided suitable data, the risk of HIV acquisition was similar in pregnant, postpartum, and non-pregnant/non-postpartum women. Finally, among African women, the risk of MTCT was 2.9-fold higher during the postpartum period among those who had recently acquired HIV than among those with chronic HIV infection, and 2.3-fold higher during the pregnancy/postpartum periods combined.

**What Do These Findings Mean?:**

These results suggest that women living in regions where HIV infection is common are at high risk of acquiring HIV infection during pregnancy and the postpartum period and that mothers who acquire HIV during pregnancy or postpartum are more likely to pass the infection on to their offspring than mothers with chronic HIV infections. However, the small number of studies included in this meta-analysis and the use of heterogeneous research methodologies in these studies may limit the accuracy of these findings. Nevertheless, these findings have important implications for the global plan to eliminate HIV infections in children. First, they suggest that women living in regions where HIV infection is common should be offered repeat HIV testing (using sensitive methods to enhance early detection of infection) during pregnancy and in the postpartum period to detect incident HIV infections, and should be promptly referred to HIV care and treatment. Second, they suggest that prevention of HIV transmission during pregnancy and postpartum should be prioritized, for example, by counseling women about the need to use condoms to prevent transmission during this period of their lives.

**Additional Information:**

Please access these websites via the online version of this summary at http://dx.doi.org/10.1371/journal.pmed.1001608.

- Information is available from the US National Institute of Allergy and Infectious Diseases on HIV infection and AIDS
- NAM/aidsmap provides basic information about HIV/AIDS and summaries of recent research findings on HIV care and treatment
- Information is available from Avert, an international AIDS charity, on many aspects of HIV/AIDS, including information on children and HIV/AIDS and on the prevention of mother-to-child transmission of HIV (in English and Spanish)
- The 2013 UNAIDS World AIDS Day Report provides information about the AIDS epidemic and efforts to halt it; the 2013 UNAIDS Progress Report on the Global Plan provides information on progress towards eliminating new HIV infections among children; the UNAIDS Believe it. Do it website provides information about the campaign to support the UNAIDS global plan
- Personal stories about living with HIV/AIDS, including stories from young people infected with HIV, are available through Avert, NAM/aidsmap, and Healthtalkonline

## Introduction

Antenatal HIV testing is essential to identify HIV-infected women who need to start antiretrovirals (ARVs) both to decrease risk of mother-to-child HIV transmission (MTCT) and improve maternal health. As part of this process, women who test HIV negative during antenatal screening may feel reassured that neither they nor their infants are at risk for HIV. However, HIV may be acquired during pregnancy and postpartum and would not be detected unless repeat HIV testing is conducted. Despite guidelines recommending repeat HIV testing during the third trimester or at delivery in settings where the HIV epidemic is generalized [1],[2], repeat testing is rarely implemented or documented [3],[4]. The lack of retesting during pregnancy and postpartum represents a missed opportunity to identify women who have recently acquired HIV infection and have an increased risk of MTCT because of their high HIV viral loads during incident infection, and to initiate ARVs for prevention of mother-to-child HIV transmission (PMTCT) among HIV-infected women who did not access antenatal care and were not tested during pregnancy [5].

Several individual studies suggest that the incidence of HIV infection during pregnancy and the postpartum period is high, with some suggesting increased incidence among pregnant/postpartum women compared to non-pregnant women [3],[5],[6]. However, differences in antenatal and postpartum follow-up, study designs, assays used to detect infection, and underlying HIV prevalence have made it difficult to compare findings between studies. Potential mechanisms for increased susceptibility to HIV during pregnancy and postpartum include both biological and behavioral characteristics unique to this period [6]. If the risk of HIV acquisition is increased during the pregnancy and/or postpartum periods, this higher risk may translate to a substantial cumulative period of risk for women in areas where fertility rates and HIV prevalence are high.

While a systematic review conducted in 1992 by Dunn et al. quantified risk of MTCT through breastfeeding among women infected postnatally, risk of in utero or intrapartum transmission among women who acquired HIV during pregnancy was not included [7]. In addition, the review was conducted prior to the implementation of ARV prophylaxis for PMTCT and was unable to characterize risk of MTCT among mothers with incident HIV infection in the era of PMTCT ARVs. Thus, the relative contribution of maternal HIV acquisition during pregnancy or postpartum and use of maternal PMTCT ARVs is unknown.

In 2012, the World Health Organization announced a global plan to eliminate MTCT by 2015 that includes reducing HIV incidence among women and MTCT rates, and increasing uptake of maternal and infant ARVs for PMTCT [8]. However, achievement of these objectives will be hindered without a greater understanding of the risk of HIV infection in pregnant and postpartum populations, and the subsequent risk of MTCT both in the presence and absence of PMTCT ARVs. We performed a systematic review and meta-analysis to synthesize and compare the risk of HIV acquisition during pregnancy and the postpartum period, and risk of MTCT among women with incident HIV infection.

## Methods

### Systematic Review and Article/Abstract Selection

**Figure 1:**
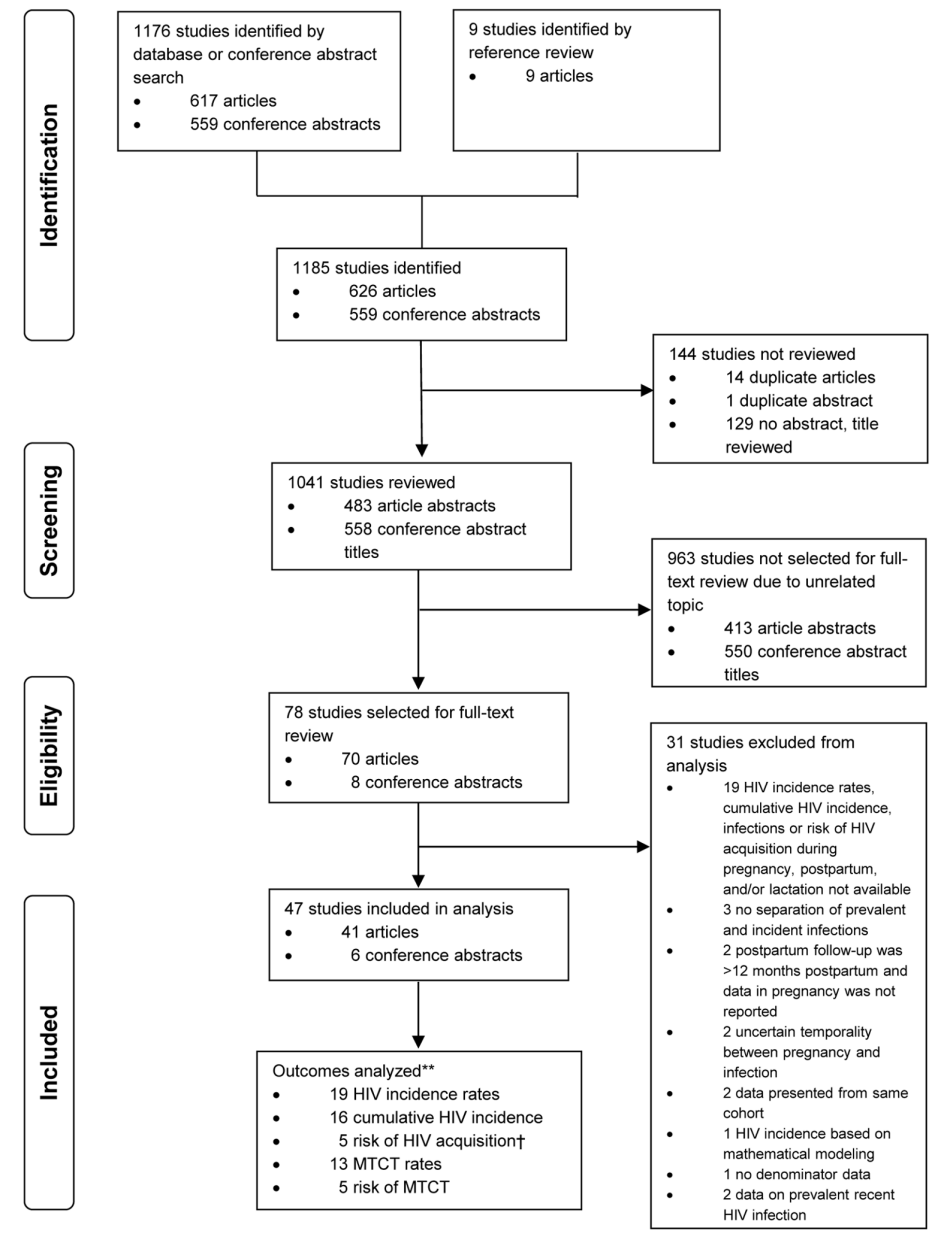
Flowchart of studies reviewed and included in meta-analyses. **Not mutually exclusive. †One study excluded that reported relative risk rather than hazard ratio.

We conducted a systematic review for all peer-reviewed published articles and conference abstracts on recent HIV infection during pregnancy or through 12 months postpartum (Figure 1). PubMed and Embase databases were searched from January 1, 1980, through October 31, 2013, to identify articles to review using combinations of the following terms: HIV infections, HIV seropositivity, pregnancy, human, incidence, acquisition, and acute. For PubMed (Medline) our search strategy was the following: (HIV infections[mh] or hiv-seropositivity[mh]) AND pregnancy[mh] AND human[mh] AND (incidence[mh] OR incidence[ti] OR acquisition[ti] OR acute[ti]). Our search strategy for Embase (excluding Medline results) was: ‘Human immunodeficiency virus infection’/exp AND ‘pregnancy’/exp AND (‘incidence’/exp OR ‘incidence’:ti OR ‘acquisition’:ti OR ‘acute’:ti) AND [embase]/lim NOT [medline]/lim. Titles of abstracts from the 19th and 20th Conference on Retroviruses and Opportunistic Infections (2011 and 2012), 6th and 7th International AIDS Society Conference (2011 and 2013), and 19th International AIDS Conference (2012) were also searched using the terms pregnancy, postpartum, and lactating. Titles of articles without abstracts were reviewed for consideration of full-text review; however, duplicate abstracts were not considered for full-text review. Articles were included in full-text review if the abstract or title mentioned HIV incidence, HIV seroconversion, or recent HIV infection during pregnancy, the postpartum period, or lactation. Review articles and articles on unrelated topics were excluded from full-text review. Titles of references cited in studies selected for full-text review were also evaluated for relevance and full-text review. Full-text articles and conference abstracts were excluded from analyses if (1) HIV incidence rates; cumulative HIV incidence; risk of HIV acquisition during pregnancy, postpartum, and/or during lactation; risk of MTCT during incident infection; or MTCT rates among women with incident infections were not reported and could not be calculated; (2) HIV infection preceded pregnancy; (3) HIV incidence estimates were based on mathematical modeling; (4) postpartum follow-up was >12 mo postpartum and HIV incidence during pregnancy was not reported; or (5) HIV prevalence and incidence could not be distinguished from one another. Additionally, conference abstracts that were subsequently published as articles were excluded, studies reporting results from the same cohort were compared and only the article with the most detailed data on incidence and follow-up was included, and articles in English were included if some or all of the data were also published in a different journal and in a language other than English. All other non-English articles/abstracts were considered for full-text review, but either were found to not be relevant based on review of English abstracts and titles, or did not have available abstracts to review. The combined search of articles and conference abstracts resulted in 1,176 studies for review: 626 articles and 559 conference abstracts. An additional nine articles were included for review from references cited in these articles, for a total of 1,185 studies identified to review. Among these, 78 studies (70 articles and eight conference abstracts) were identified for full-text review.

### Data Extraction and Statistical Analysis

Two independent reviewers (A. L. D. and A. W.) reviewed full-text articles for inclusion and exclusion and abstracted data on the following outcomes occurring during pregnancy and/or postpartum periods: (1) HIV incidence rates; (2) cumulative incidence of HIV; (3) risk of HIV acquisition (hazard ratio [HR]); (4) risk of MTCT (odds ratio [OR]); and (5) MTCT rate. Variables extracted for analysis included sample size, number of incident HIV infections, number of women at risk of incident infection, person-time of follow-up, HIV incidence rates, cumulative HIV incidence, number of infant infections, assay used to detect incident infections, retesting and/or follow-up intervals, postpartum and lactation status, and study location. All postpartum follow-up time was restricted to 12 months postpartum unless women were reported to be lactating; there was no restriction on follow-up time for lactating women. Articles that did not report maximum postpartum follow-up time but presented a mean or median postpartum follow-up ≤12 months were also included in the analysis. Lactating women were combined with postpartum women for the meta-analysis; however, a separate subgroup analysis of postpartum women who were defined as lactating versus postpartum was also conducted.

#### Incidence rates and cumulative incidence

Incidence rates are reported as the number of new infections per 100 person-years; rates were calculated if data were available but not presented as an incidence rate. Cumulative incidence was calculated as the number of new infections per number at risk for studies with repeat testing. Cumulative incidence based on cross-sectional testing of HIV-positive samples using assay algorithms designed to detect incident infections was included as presented in the article or abstract. Assays included in the cumulative incidence meta-analysis included serological testing algorithm for recent HIV seroconversion (STARHS) using BED capture enzyme immunoassay or bioMerieux Vironosticka less sensitive enzyme immunoassay, avidity index, nucleic acid amplification tests, and p24 antigen tests. Studies presenting annual cumulative incidence were summarized for the meta-analysis by calculating a weighted incidence based on sample size. We calculated 95% CIs if they were not presented in the study. The Poisson distribution was used to calculate 95% CIs for incidence rates using person-time denominators, and the binomial distribution was used to calculate 95% CIs for cumulative incidence using individuals as the denominator. 95% CIs were calculated using the weighted estimate if annual cumulative incidence was calculated by weighting. Authors were contacted if additional information was required to calculate incidence rates or cumulative incidence.

#### Pooled summary statistics and plots

Meta-analyses were conducted to summarize cumulative HIV incidence and incidence rates of HIV during pregnancy and postpartum, MTCT rates among women with incident HIV infections, the association between pregnancy/postpartum periods and HIV acquisition, and the association between incident maternal HIV infection during pregnancy/postpartum periods and MTCT. Pooled HRs were calculated to summarize the association between pregnancy and postpartum periods and HIV acquisition if HRs were presented, and pooled ORs were used to summarize the association between incident infection during pregnancy/postpartum periods and MTCT. Fixed effects models were constructed to pool HIV incidence rates, cumulative incidence, MTCT rates, HRs, and ORs; models were tested for heterogeneity using the *I* ^2^ statistic. Models pooling HIV incidence rates, MTCT rates, and HRs for HIV acquisition were a priori stratified by pregnancy, postpartum period, or pregnancy and postpartum periods combined. Significant heterogeneity was detected in all models, and the models were reconstructed with random effects using estimates of heterogeneity from the Mantel-Haenszel model to calculate pooled measures. We conducted both a priori and post hoc analyses to identify sources of heterogeneity. Pooled incidence rates during pregnancy and the postpartum period for studies conducted in Africa were a priori stratified by region (south, southeast, east, central, and west; in descending order of HIV prevalence) as a strategy to adjust for differences in HIV prevalence between these regions. We compared cumulative incidence detected by assay algorithms versus repeat testing and MTCT risk by use of maternal or infant ARV prophylaxis through additional stratified analysis. We also conducted post hoc stratified analysis of MTCT rates, MTCT risk, and cumulative incidence by study location (Africa or outside of Africa), and multivariate meta-regression for cumulative incidence by study location and testing algorithm. Forest plots of HIV incidence rates, cumulative HIV incidence, HRs, ORs, and MTCT rates were generated for individual articles, pooled subgroup estimates (when indicated), and pooled overall estimates. Meta-regression was used to compare log-transformed incidence, log-transformed cumulative incidence, MTCT rates, ORs, and HRs between subgroups and in multivariate models. Statistical analysis was performed using Stata version 13.1 (StataCorp).

The Newcastle-Ottawa Scale was used to assess the quality of the studies included in the meta-analyses that compared MTCT risk or risk of maternal HIV acquisition between groups; this scale ranges from 0 to 9 and assesses study quality based on study characteristics for selection of the cohort, comparability of exposed and unexposed groups, and outcome ascertainment. The quality of studies for studies with comparison groups was not compared statistically since quality scoring adds subjective bias to the results unless individual score components are used in the meta-regression, and adding several covariates for individual score components would result in over-stratification [9],[10]. Two independent reviewers (A. L. D. and A. W.) scored each study, and the average score for all studies, stratified by outcome, was calculated [11].

## Results

### Study Selection for Review

Among the 1,176 studies identified, 78 were selected for further review of the full-text article or conference abstract, and 47 were included in the analysis (41 articles and six conference abstracts) (Figure 1). Characteristics of studies included in the meta-analysis and data summaries are presented in Table 1. Studies were identified from six continents: 35 from Africa; four each from Asia, North America, and South America; and one each from Australia and Europe. Only ten studies comparing MTCT risk (*n* = 5) or risk of maternal HIV acquisition (*n* = 5) between groups were eligible for evaluation by the Newcastle-Ottawa Scale. Studies evaluated by the Newcastle-Ottawa Scale received a mean score of 7.4 for studies comparing risk of MTCT and a mean score of 8.2 for studies comparing risk of maternal HIV acquisition, indicating that the quality of included studies was high; individual Newcastle-Ottawa Scores for MTCT and risk of maternal HIV acquisition are shown in Table 1.

**Table 1:** Study characteristics and data included in meta-analyses and data summaries.

Study Location	First Author, Year [Reference]	Study Years	Country	Methodology	Sample Size or Person-Years	Incidence Measure (Pregnancy or Postpartum)	Repeat Testing Intervals	HIV Acquisition	MTCT Measures (Pregnancy or Postpartum)^a^	Newcastle-Ottawa Score (Analysis)
**Africa**										
**South**	Bernasconi, 2010 [53]	2004–2006	Swaziland	Assay	*n* = 3,029	CI (pregnancy)	—	—	—	—
	Boly, 2011 [54]	2010	Namibia	Retesting	*n* = 196	CI (pregnancy)	—	—	—	—
	Hargrove, 2008 [14]	1997–2000	Zimbabwe	Assay	*n* = 6,829	CI (postpartum)	—	—	—	—
	Humphrey, 2006 [46]	1997–2001	Zimbabwe	Prospective cohort	7,763 PY	IR (postpartum)	Postpartum (quarterly/biannually until 12 mo postpartum)	—	—	—
	Humphrey, 2010 [21]	1997–2000	Zimbabwe	Prospective cohort	*n* = 334	—	—	—	Rate and risk (pregnancy)	7 (MTCT)
	Kharsany, 2010 [55]	2007–2008	South Africa	Assay	*n* = 467	CI (pregnancy)	—	—	—	—
	Kieffer, 2011 [56]	2008–2009	Swaziland	Prospective cohort	346 PY	IR (pregnancy)	—	—	—	—
	Lu, 2011 [16]	Not reported	Botswana	Retesting	*n* = 417 (incidence), *n* = 17 (MTCT)	CI (pregnancy and postpartum)	—	—	Rate (pregnancy and postpartum)	—
	Mbizvo, 2001 [57]	1991–2005	Zimbabwe	Prospective cohort	1,093 PY	IR (pregnancy and postpartum)	Postpartum (12 mo postpartum)	—	—	—
	Moodley, 2009 [3]	2006–2007	South Africa	Retesting	679 PY		Pregnancy (36–40 wk gestation)	—	—	—
	Moodley, 2011^b^ [5]	2005–2007	South Africa	Prospective cohort	1,946 PY, *n* = 39	IR (pregnancy and postpartum)	Pregnancy and postpartum (≤6 mo antenatally and 12 mo postpartum)	—	Rate and risk (pregnancy and postpartum combined)	7 (MTCT)
	Morrison, 2007^c^ [42]	1994–2004	Zimbabwe	Prospective cohort	2,004 PY, *n* = 4,415	IR (pregnancy and postpartum)	Pregnancy (≤24 mo after enrollment)	Pregnancy and postpartum	—	9 (HIV acquisition)
	Munjoma, 2010 [58]	2002–2008	Zimbabwe	Prospective cohort	298 PY	IR (pregnancy)	—	—	—	—
	Rehle, 2007 [13]	2005	South Africa	Assay	*n* = 135	CI (pregnancy)	—	—	—	—
	Reid, 2010 [59]	2003–2007	3 African countries	Prospective cohort	*n* = 228	—	—	Pregnancy	—	8 (HIV acquisition)
	Rollins, 2002 [60]	2000	South Africa	Assay	*n* = 418	—	—	—	—	—
	Wand, 2011 [39]	2002–2005	South Africa	Prospective cohort	*n* = 2,523	—	—	Pregnancy	—	8 (HIV acquisition)
**Southeast**	De Schacht, 2011 [61]	Not reported	Mozambique	Prospective cohort	226 PY	IR (pregnancy)	—	—	—	—
	Gay, 2010 [62]	2000–2004	Malawi	Assay	*n* = 2,327	CI (pregnancy)	—	—	—	—
	Hira, 1990 [20]	1987	Zambia	Retesting	*n* = 1,954 (incidence), *n* = 19 (MTCT)	CI (pregnancy and postpartum)	Postpartum (12 mo postpartum)	—	Rate (postpartum)	—
	Mugo, 2011 [38]	2004–2007	7 African countries	Prospective cohort	231 PY, *n* = 1,085	IR (pregnancy)	—	Pregnancy	—	9 (HIV acquisition)
	Taha, 1998 [45]	Recruited 1990, 1993	Malawi	Prospective cohort	338 PY	IR (pregnancy)	—	—	—	—
**Central**	Colebunders, 1988 [19]	Not reported	Zaire	Case series	*n* = 3	—	—	—	Rate (postpartum)	—
**East**	Braunstein, 2011^c^ [63]	2006–2009	Rwanda	Prospective cohort	625 PY, *n* = 397	IR (pregnancy and postpartum)	Postpartum (2 y after enrollment)	Pregnancy and postpartum	—	7 (HIV acquisition)
	Gray, 2005^c^ [6]	1994–1999	Uganda	Prospective cohort	4,040 PY	IR (pregnancy and postpartum)	Postpartum (until lactation stopped)	—	—	—
	Keating, 2012 [43]	2009	Malawi	Retrospective cohort	275 PY	IR (pregnancy)	—	—	—	—
	Kinuthia, 2010 [64]	Not reported	Kenya	Retesting	779 PY	IR (pregnancy)	Pregnancy (6 wk postpartum)	—	—	—
	Leroy, 1994 [65]	1988–1992	Rwanda	Prospective cohort	204 PY	IR (postpartum)	Postpartum (quarterly until 12 mo postpartum)	—	—	—
	Tabu, 2013 [4]	2012	Uganda	Retesting	312 PY	IR (pregnancy)	—	—	—	—
	Van de Perre, 1991 [22]	1988–1991	Rwanda	Prospective cohort	*n* = 12	—	—	—	Rate (pregnancy and postpartum)	—
	Wawer, 1999 [66]	1994	Uganda	Prospective cohort	1,280 PY	IR (pregnancy and postpartum)	Postpartum, retested 8 mo postpartum (mean)	—	—	—
	Wolday, 2007 [67]	1995–2003	Ethiopia	Assay	*n* = 6,394	CI (pregnancy)	—	—	—	—
**West**	Imade, 2012 [68]	2010–2012	Nigeria	Assay	235 PY	IR (pregnancy)	—	—	—	—
	Kim, 2010 [12]	1998–2004	Côte d'Ivoire	Assay	*n* = 10,616	CI (pregnancy)	—	—	—	—
	Traore, 2012 [69]	2010–2011	Burkina Faso	Prospective cohort	126 PY	IR (pregnancy)	—	—	—	—
**Asia**	Duan, 2010 [70]	2004–2008	China	Assay	*n* = 84,144	CI (pregnancy)	—	—	—	—
	Liang, 2009 [15]	2000–2008	China	Retrospective cohort	*n* = 106	—	—	—	Rate (postpartum)	—
	Roongpisuthipong, 2001 [24]	1992–1994	Thailand	Retesting	*n* = 15	—	—	—	Rate and risk (pregnancy)	7 (MTCT)
	Saphonn, 2005 [71]	1999–2002	Cambodia	Assay	*n* = 19,467	—	—	—	—	—
**North America**	Birkhead, 2010 [25]	2002–2006	United States (New York)	Prospective cohort	*n* = 41	—	—	—	Rate and risk (pregnancy)	9 (MTCT)
	Nesheim, 2005 [72]	1990–1998	United States (Georgia)	Assay	*n* = 48,018	CI (pregnancy)	—	—	—	—
	Nesheim, 2007 [26]	2001–2005	United States	Retesting	*n* = 4	—	—	—	Rate (pregnancy)	—
	Singh, 2012 [18]	2005–2010	United States	Surveillance cohort	*n* = 124	—	—	—	Rate and risk (pregnancy)	7 (MTCT)
**South America**	de Freitas, 2005 [73]	1991–2002	Brazil	Assay	*n* = 4,327	CI (pregnancy)	—	—	—	—
	Pando, 2011 [74]	2006–2008	Argentina	Assay	*n* = 8,560	CI (pregnancy)	—	—	—	—
**Australia**	Palasanthiran, 1993 [17]	1984–1990	Australia	Retrospective cohort	*n* = 11	—	—	—	Rate (postpartum)	—
**Europe**	Tovo, 1991 [23]	Not reported	Italy	Prospective cohort	*n* = 10	—	—	—	Rate (pregnancy)	—

a MTCT rates measured as proportions, and risk measured as ORs.

b Antenatal and postpartum periods combined.

c Postpartum women were defined as lactating.

CI, cumulative incidence; IR, incidence rate; PY, person-years.

### Incidence Rates during Pregnancy and Postpartum

**Figure 2:**
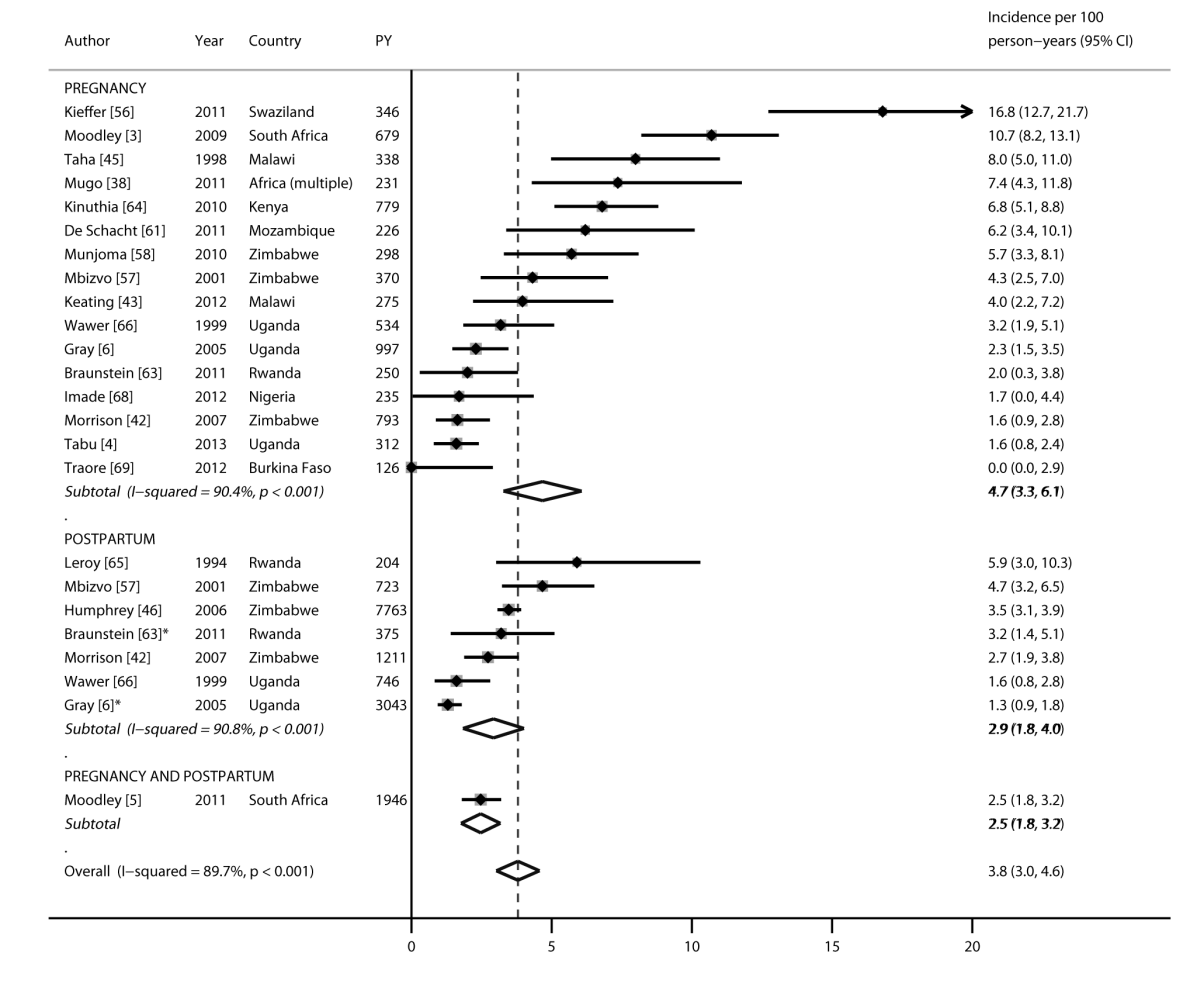
Forest plot of HIV incidence rates, by pregnancy and postpartum status. PY, person-years. *Defined as lactating.

**Figure 3:**
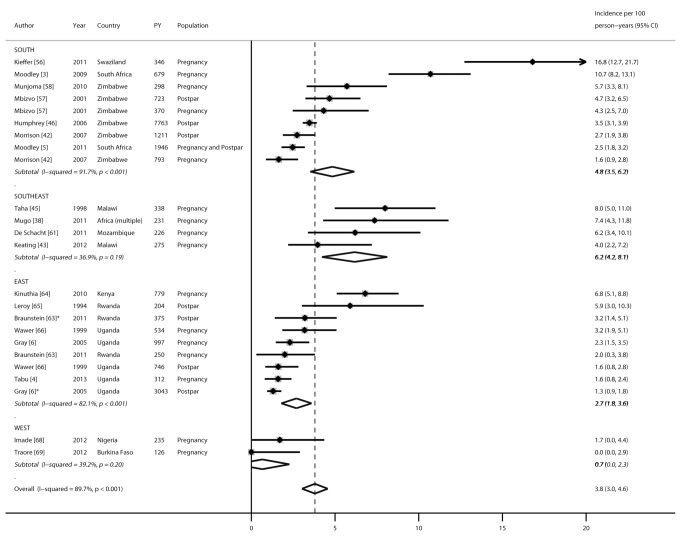
Forest plot of HIV incidence rates during pregnancy and postpartum, by African region. PY, person-years. *Defined as lactating.

Nineteen studies reported HIV incidence rates during pregnancy and/or the postpartum period: 16 during pregnancy, seven during the postpartum period, and one during pregnancy and postpartum periods combined (Figure 2), representing 19 cohorts and 22,803 person-years in total. All incidence rates were from publications reported from sub-Saharan Africa. Incidence rates during pregnancy ranged from 0 to 16.8 per 100 person-years, with a pooled incidence rate of 4.7 (95% CI 3.3–6.1). During the postpartum period, the pooled incidence rate was 2.9 (95% CI 1.8–4.0) and not significantly different from the pooled incidence rate during pregnancy (4.7, 95% CI 3.3–6.1; *p* = 0.18). After combining results from all studies, the pooled incidence during pregnancy and/or the postpartum period was 3.8 (95% CI 3.0–4.6). In subgroup analyses separating postpartum women who were defined as lactating versus postpartum, stratified pooled incidence rates among women defined as lactating (2.0, 95% CI 0.2–3.8) or postpartum (3.2, 95% CI 2.2–4.2) were not significantly different from that of the overall postpartum population combining these groups. Incidence rates during pregnancy and/or postpartum were also stratified by African region (Figure 3). Pooled incidence was highest in southeast Africa (6.2, 95% CI 4.2–8.1), followed by south Africa (4.8, 95% CI 3.5–6.2), east Africa (2.7, 95% CI 1.8–3.6), and west Africa (0.7, 95% CI 0–2.3); the pooled incidence rate was significantly lower in east Africa than in southeast Africa (*p* = 0.04), but there were no other significant differences by region.

### Cumulative HIV Incidence during Pregnancy and Postpartum

**Figure 4:**
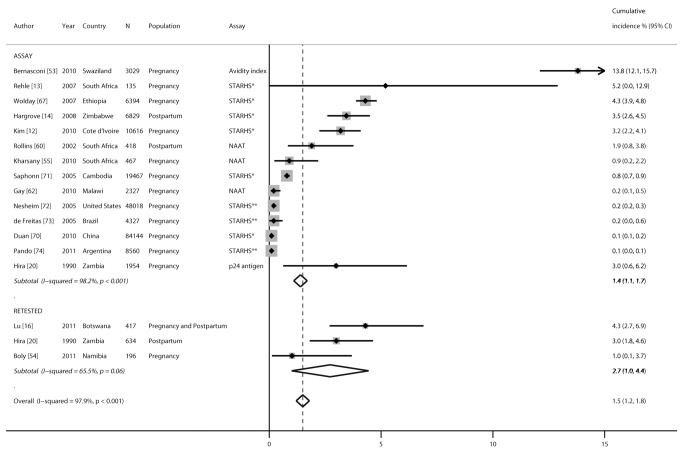
Forest plot of cumulative incidence of incident HIV infection during pregnancy and postpartum, by testing algorithm. The retested cumulative incidence category is calculated as the number of new infections per number at risk for studies, and the assay cumulative incidence category is calculated based on cross-sectional testing of HIV-positive samples using assay algorithms designed to detect incident infections; cumulative incidence expressed as percent for both retested and assay categories. NAAT, nucleic acid amplification test; STARHS*, STARHS using BED capture enzyme immunoassay; STARHS**, STARHS using bioMerieux Vironosticka less sensitive enzyme immunoassay.

Cumulative HIV incidence during pregnancy was reported in 16 studies, during the postpartum period in three studies, and during the combined pregnancy/postpartum period in one study (Figure 4). Thirteen studies reported cumulative incidence based on assay-specific algorithms from cross-sectional testing, two studies were based on repeat testing, and one study reported both assay and repeat testing results during pregnancy, delivery, or postpartum. Incident infections from assays were detected using an avidity index, STARHS, p24 antigen detection, or nucleic acid amplification tests. The estimate of cumulative incidence using BED capture enzyme immunoassay as the assay for STARHS was adjusted to account for assay sensitivity and specificity in the studies by Kim et al. [12], Rehle et al. [13], and Hargrove et al. [14], as reported in the articles. Pooled cumulative incidence was 1.4% (95% CI 1.1%–1.7%) when infections were detected by assay algorithms from cross-sectional testing and 2.7% (95% CI 1.0%–4.4%) among populations retested (*p* = 0.4). Cumulative incidence was significantly higher in African countries (3.6%, 95% CI 1.9%–5.3%) than in non-African countries (0.3%, 95% CI 0.1%–0.4%; *p*<0.001) and remained significant (*p*<0.001) in a meta-regression model including study location (Africa or outside of Africa).

### Association between Pregnancy and/or Postpartum Period and Risk of HIV Acquisition

**Figure 5:**
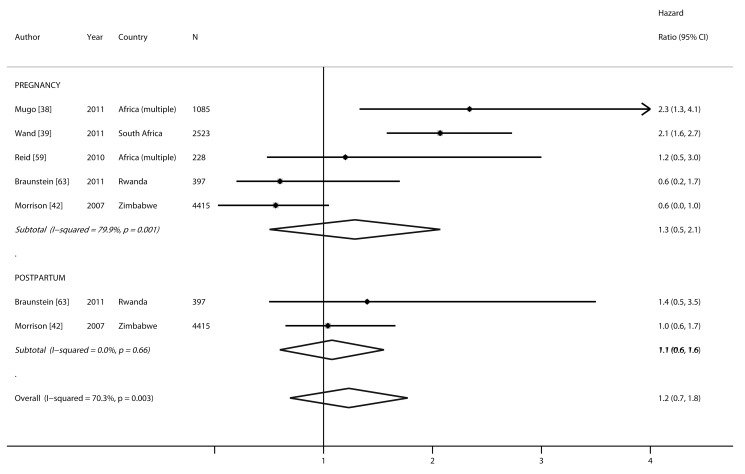
Forest plot of risk of HIV acquisition, by pregnancy and postpartum status.

Two studies compared the HIV incidence rate during pregnancy and/or postpartum, separately, to the incidence rate of non-pregnant/non-lactating women. Three studies reported HIV incidence only for pregnant women compared to non-pregnant women. Risk of HIV acquisition among pregnant (pooled HR 1.3, 95% CI 0.5–2.1) and postpartum women (pooled HR 1.1, 95% CI 0.6–1.6) was not significantly different from non-pregnant/non-lactating women (*p* = 0.92). A non-significantly higher risk of HIV acquisition was also observed when data from the pregnancy and postpartum periods were combined and compared to non-pregnant/non-lactating women (pooled HR 1.2, 95% CI 0.7–1.8) (Figure 5).

### Mother-to-Child Transmission among Women with Incident HIV during Pregnancy or Postpartum

**Figure 6:**
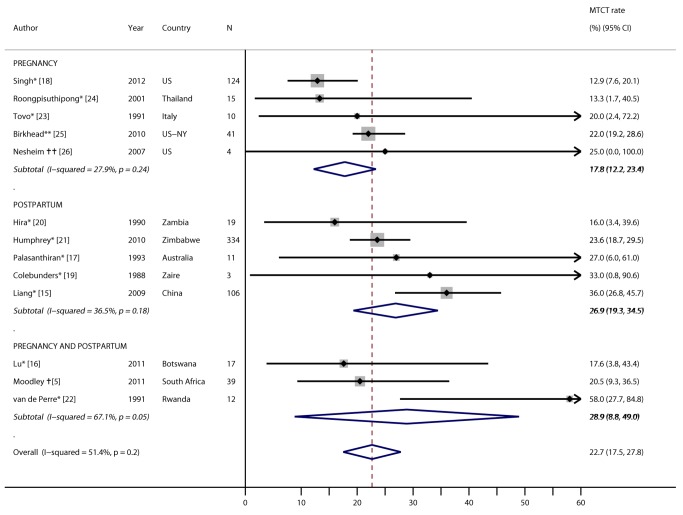
Forest plot of mother-to-child HIV transmission rates among women with incident infection during pregnancy/postpartum. *No PMTCT ARVs. **Unspecified ARV regimen. †Single-dose nevirapine if maternal CD4 count >200 cells/mm^3^ and ART for women with CD4 count ≤200 cells/mm^3^. ††Zidovudine and nevirapine regimens for women and infants.

MTCT rates among women with incident infections detected during pregnancy or postpartum were presented in 13 studies, representing 11 countries and four continents (Figure 6). Five of these studies identified women who became infected during the postpartum period (three studies documented infections following postnatal blood transfusions), three reported MTCT rates among women infected during pregnancy or postpartum, and five reported MTCT rates among women infected during pregnancy. Ten studies provided no PMTCT: four studies provided no PMTCT because of cross-sectional, simultaneous ascertainment of maternal and infant HIV infections or testing after infant exposure to HIV had stopped [15]–[18], and six studies were conducted prior to PMTCT implementation at the study sites [19]–[24]. Three studies documented provision of PMTCT ARVs [5],[25],[26]: one used single-dose nevirapine for women with CD4 count >200 cells/mm^3^ and antiretroviral therapy (ART) for women with CD4 count ≤200 cells/mm^3^, one used zidovudine and nevirapine regimens for women and infants, and the last did not specify ARV regimen. MTCT rates across all studies ranged from 12.9% in the United States to 58.0% in Rwanda, with a pooled rate of 22.7% (95% CI 17.5%–27.8%). A similar MTCT rate (23.5%) resulted from the pooled analysis of the subset of these studies that did not use ARVs (*p* = 0.70); MTCT rates also did not differ by African versus non-African countries (23.6% versus 22.0%, respectively; *p* = 0.49). The pooled MTCT rate among women with incident infection during pregnancy was 17.8% (95% CI 12.2%–23.4%), compared to 26.9% (95% CI 19.3%–34.5%) among women with incident infection postpartum (*p* = 0.10).

**Figure 7:**
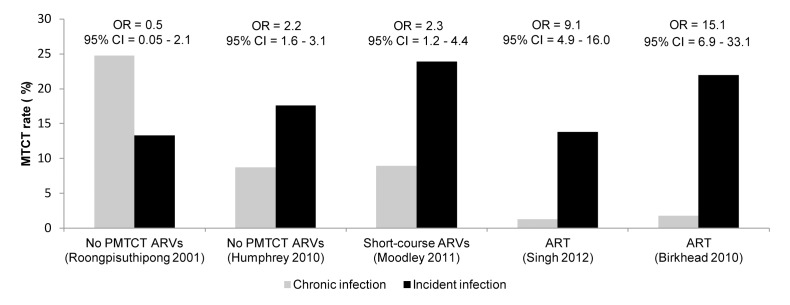
Effect of incident maternal HIV infection and antiretroviral prophylaxis on mother-to-child HIV transmission. Incident infection defined as maternal HIV acquisition during pregnancy or postpartum; chronic infection defined as established HIV infection during pregnancy or postpartum. MTCT rates and ORs are derived from the studies indicated in parentheses; PMTCT ARVs represent the regimens available during the studies. For Moodley [5], women with CD4 count >200 cells/mm^3^ were eligible to receive ART; all other women received single-dose nevirapine regimens. For Singh [18], women were assumed to receive ART as per national guidelines in place during the study.

**Figure 8:**
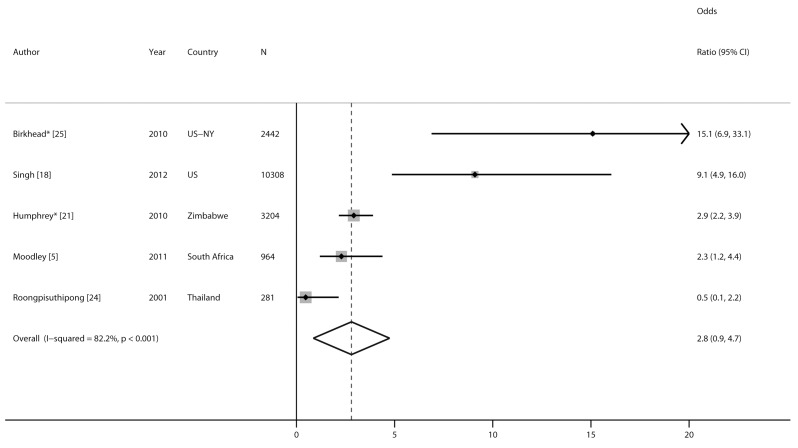
Forest plot of risk of mother-to-child HIV transmission and maternal HIV infection status.

Five studies compared risk of MTCT among women with incident versus chronic HIV infection (Figures 7 and 8). MTCT risk was 9- to 15-fold higher among US women who seroconverted during pregnancy than among women with chronic HIV infection, because of effective early ART treatment of chronically HIV-infected mothers. In African cohorts, compared to women with chronic HIV, MTCT risk was 2- to 3-fold higher among women with incident HIV infection in the postpartum period (pooled OR 2.9, 95% CI 2.2–3.9) or in the pregnancy and postpartum periods combined (pooled OR 2.3, 95% CI 1.2–4.4). The pooled OR for MTCT with incident HIV versus chronic HIV among mothers was 2.8 (95% CI 0.9–4.7) (Figure 8); the pooled OR after adjusting for maternal or infant ARV prophylaxis was 5.6 (95% CI 0.1–311.5).

## Discussion

In this meta-analysis we found that HIV incidence rates were high among pregnant and postpartum women, with pooled incidence rates of 4.7 per 100 person-years among pregnant women and 2.9 per 100 person-years among postpartum women. In our pooled analysis, we did not find a significantly higher risk of HIV infection among pregnant or postpartum women compared to non-pregnant women. The pooled MTCT rate was high, 22.7%, among pregnant and postpartum women with incident HIV infection, and risk of MTCT was 2.8-fold higher among these women compared to women with chronic HIV infection. Pooled MTCT rate was non-significantly higher among women with postpartum versus antenatal incident infections (26.9% versus 17.8%, respectively).

The highest HIV incidence estimates during pregnancy/postpartum were >10 per 100 person-years and were from South Africa and Swaziland, countries with high HIV prevalence (>15%) [27] and in which high incidence has been noted in non-pregnant adults [28],[29]. The pooled incidence rates we observed are comparable to, or higher than, those of non-pregnant “high risk” individuals, including female sex workers (2.7 per 100 person-years), HIV-discordant couples (2.0 to 3.6 per 100 person-years) from south and east Africa, and men who have sex with men (6.1 per 100 person-years) from North and South America [30]–[35]. Studies of discordant couples involve a known HIV-infected partner, which would be expected to result in higher HIV incidence than in pregnant women with unknown partner status, the majority of whom presumably have HIV-uninfected partners. However, discordant couples in cohort studies, by virtue of knowing their status, have reinforced messages to use condoms, access ART treatment, and decrease risk, leading to lower HIV incidence. Similarly, studies in female sex workers have noted dramatic reductions in HIV incidence following initial enrollment in cohort studies [36],[37], because of either increased HIV preventive measures or selective inclusion of relatively resistant individuals in the cohort.

While single studies have noted increased risk of HIV acquisition during pregnancy/postpartum compared to non-pregnant women [38],[39], the pooled estimate did not suggest that pregnancy or the postpartum period significantly elevated risk. However, it is difficult to design a study that would definitively determine the incremental risk of the pregnancy/postpartum periods compared to non-pregnant time periods because of a variety of confounding factors. One notable potential confounder is use of contraceptives in the non-pregnant/postpartum comparison group, some of which (injectable and oral contraceptives) have also been linked to higher risk of HIV acquisition [40],[41]. One study in this meta-analysis included hormonal contraception within the multivariate model and found that the effect of pregnancy on HIV acquisition risk was attenuated when potential confounders were included in the model [38], whereas another study did not observe any difference in risk when non-pregnant/non-lactating women using versus not using hormonal contraception were used as comparison groups [42]. In addition, differential risk during early versus late pregnancy may modify the association between pregnancy and HIV risk. Studies included in this meta-analysis included both early and late stages of pregnancy in their risk intervals, but did not capture associations with specific periods in pregnancy, for example, whether risk increases as hormone levels rise. Mugo et al. [38] did not observe any difference in associations, based on stage of pregnancy, but the sample size was small, limiting the power to detect effect modification by pregnancy stage. Finally, while HIV incidence was non-significantly higher during pregnancy versus non-pregnant time periods, the lower coital frequency among pregnant and postpartum African women previously reported [6],[43],[44] may indicate that pregnancy is associated with increased per-coital-act susceptibility to HIV, as has been observed in a study conducted in Uganda [6]. Thus, an alternative approach to determining the impact of the pregnancy/postpartum periods would be to pool estimates of risk per coital act, as was done in the Ugandan study, which estimated 1.4-fold higher HIV risk per coital act during pregnancy/postpartum compared to non-pregnant/non-lactating periods [6]. However, detailed data on frequency of intercourse were not provided in the studies reviewed, limiting ability to estimate risk per coital act.

Both biological and behavioral changes have been hypothesized to explain the potentially higher risk of HIV acquisition observed during pregnancy and the postpartum period. Behavioral factors were not associated with HIV acquisition in two prior studies of pregnant and postpartum women [3],[6]; however, behavior changes in male partners during the pregnancy/postpartum period have not been well characterized and could play a role in maternal HIV risk, particularly if partners increase sexual activity outside of the relationship. Pregnancy-induced physiological changes have been hypothesized to increase HIV susceptibility, through changes in systemic and mucosal immunity, disturbances in vaginal flora, and alterations of the genital mucosa. However, prospective studies to examine biological risk factors for HIV acquisition during pregnancy and postpartum have been limited. Taha et al. showed that sexually transmitted infections (gonorrhea and trichomoniasis) and other genital tract infections (bacterial vaginosis) were associated with increased risk of HIV acquisition during pregnancy and postpartum: risk was 2-fold higher for trichomonas infection during pregnancy or postpartum, and 4-fold higher for gonorrhea during pregnancy [45]. Other potential biological risk factors for HIV acquisition include vitamin A deficiency, severe anemia, and younger age [46].

Incident maternal infections during pregnancy/postpartum may increase MTCT because of high levels of maternal viral load during incident infection, low levels of passively transferred maternal antibody, and absence of PMTCT ARVs because maternal infection is initially undetected. While half of the studies included in our MTCT rate analysis were conducted prior to implementation of PMTCT ARV prophylaxis, transmission rates were similar among women receiving and not receiving ARVs. In addition, although current World Health Organization guidelines for PMTCT are shifting from short-course ARV regimens to lifelong maternal ART, short-course regimens continue to be used in many resource-limited settings [47]. Thus, our pooled MTCT rates remain relevant to pregnant and postpartum women with both undiagnosed and treated incident infections, and highlight the need to improve detection of infections and early initiation of PMTCT ARVs.

In early MTCT studies, prior to PMTCT interventions, the increased relative risk for incident versus chronic maternal infection was due to high maternal viral load and lower maternal HIV-specific immune responses in incident infection. With PMTCT ARVs, rates of transmission among women with chronic HIV decreased from ∼20%–35% to 1%–5%. Since incident maternal HIV is typically detected weeks after infection, there is a relative delay in the initiation and effect of ARVs, amplifying the relative risk in incident versus chronic MTCT rates. Thus, earlier studies comparing MTCT in incident versus chronic maternal infection noted 2- to 3-fold increased MTCT, while later studies note 6- to >15-fold increased MTCT because of differential PMTCT. A recent modeling study projected that the absolute number of MTCT events could be reduced by 28% in South Africa if HIV screening was repeated during late pregnancy or at 6-week infant immunization visits, demonstrating a greater need for identification and early treatment for women acquiring HIV during pregnancy [48].

Our systematic review and meta-analysis had several strengths. We used a broad search strategy that included peer-reviewed articles in addition to conference abstracts presented at recent HIV conferences. We also contacted authors to acquire additional data for some summary measures. Two independent reviewers evaluated full-text articles for relevance and abstraction of data. HIV incidence rates, cumulative HIV incidence, and risk of HIV acquisition were pooled separately by pregnancy and postpartum status, as well as together, to better understand risks specific to pregnancy versus the postpartum period. To complement the meta-analysis of HIV incidence, we also pooled MTCT rates among mothers with incident infection, and compared the risk of MTCT for women with incident versus chronic infection.

Our analysis is subject to limitations resulting from pooling data from studies with heterogeneous research methodologies. Incident HIV infections reported in the studies were estimated using tests that varied in sensitivity and with different intervals for follow-up testing. Variability in test performance has previously been noted to result in overestimation of incidence when lower sensitivity tests are used initially and higher sensitivity tests for subsequent tests [49]. Timing of seroconversion is also related to timing of testing; women seeking antenatal care and testing earlier in their pregnancy have more person-time and opportunity to be detected as an incident rather than chronic infection. Conversely, seroconversions early in pregnancy are not captured as incident infections if antenatal care is sought later. Another limitation of this analysis is that cumulative incidence based on repeat testing was estimated from cohorts with differing duration of follow-up and cross-sectional testing using sensitive assays. While the performance of assays for detecting recent infections has been shown to vary across HIV clades and subpopulations, a recent study suggests pregnancy does not influence performance of BED or avidity assays [50],[51]. However, there is considerable evidence that early iterations of testing algorithms for incident infection misclassified a proportion of individuals with chronic infections as incident infections (false recent rate) and prompted the World Health Organization to issue guidance on conducting assays, with specific criteria for appropriate sampling designs, sample size, and statistical analysis considerations [51]. Thus, inclusion of different assays and testing algorithms likely overestimates our pooled cumulative incidence. These differences partially explain why our estimates of pooled cumulative incidence and pooled incidence rate differ. While we were unable to adjust for national HIV prevalence at the time of the study—since HIV prevalence estimates are not available for all countries and years included in this meta-analysis—we did consider African region as a marker of HIV prevalence; however, this approach may result in residual confounding. In addition, none of the included studies were primarily designed to estimate HIV incidence in pregnancy and the postpartum period; most studies excluded pregnant women from initial study participation, and report HIV incidence during pregnancy and postpartum as a secondary research objective, limiting generalizability. Finally, the number of studies included in our meta-regression models was small; therefore, the models may lack power to detect associations and are unable to ascertain multiple potential sources of confounding.

In conclusion, HIV incidence among pregnant and postpartum populations was high in this meta-analysis and may substantially increase risk of MTCT. Our results have several implications for antenatal care/PMTCT programs. First, women in high prevalence settings should be offered repeat HIV testing to detect incident infections and to diagnose women in the postpartum period who did not receive antenatal care. This approach is beneficial because it detects maternal HIV infection while women are accessing the health care system and prompts referral to appropriate HIV care and treatment. While specific recommendations regarding ARV regimens for women with incident infection do not currently exist, maternal ART during pregnancy is likely the best option given high maternal viral loads during incident infection. Second, there is a need for wider distribution of more sensitive HIV tests, such as the fourth generation rapid tests, to enhance early detection of incident HIV. These more sensitive assays, which detect both HIV antibodies and HIV p24 antigen, can reduce the number of women who have early HIV infection and are incorrectly classified as HIV negative. Third, since pregnant and postpartum women are a vulnerable population at risk of HIV and sexually transmitted infections, they should receive continued counseling on the need for condoms to prevent transmission during this time. Pregnant and postpartum populations should also be considered, and included early in the process, of developing and evaluating female-controlled prevention methods, such as microbicides, for safety and efficacy [52].

## Supporting Information

Checklist S1 **PRISMA checklist.**
(DOC)

## Abbreviations

ART: antiretroviral therapy
ARV: antiretroviral
HR: hazard ratio
MTCT: mother-to-child HIV transmission
OR: odds ratio
PMTCT: prevention of mother-to-child HIV transmission
STARHS: serologic testing algorithm for recent HIV seroconversion
